# The Applications of Finite Element Analysis in Proximal Humeral Fractures

**DOI:** 10.1155/2017/4879836

**Published:** 2017-09-10

**Authors:** Yongyu Ye, Wei You, Weimin Zhu, Jiaming Cui, Kang Chen, Daping Wang

**Affiliations:** ^1^Shenzhen Second People's Hospital, 3002 Sungang West Rd, Shenzhen, Guangdong Province 518035, China; ^2^Peking Union Medical College and Chinese Academy of Medical Sciences, No. 9, Dongdan San Tiao, Dongcheng District, Beijing 100730, China; ^3^Shenzhen Laboratory of Digital Orthopedic Engineering, Shenzhen Second People's Hospital, Shenzhen, Guangdong Province 518035, China; ^4^Shenzhen Key Laboratory of Tissue Engineering, Shenzhen Second People's Hospital, Shenzhen, Guangdong Province 518035, China

## Abstract

Proximal humeral fractures are common and most challenging, due to the complexity of the glenohumeral joint, especially in the geriatric population with impacted fractures, that the development of implants continues because currently the problems with their fixation are not solved. Pre-, intra-, and postoperative assessments are crucial in management of those patients. Finite element analysis, as one of the valuable tools, has been implemented as an effective and noninvasive method to analyze proximal humeral fractures, providing solid evidence for management of troublesome patients. However, no review article about the applications and effects of finite element analysis in assessing proximal humeral fractures has been reported yet. This review article summarized the applications, contribution, and clinical significance of finite element analysis in assessing proximal humeral fractures. Furthermore, the limitations of finite element analysis, the difficulties of more realistic simulation, and the validation and also the creation of validated FE models were discussed. We concluded that although some advancements in proximal humeral fractures researches have been made by using finite element analysis, utility of this powerful tool for routine clinical management and adequate simulation requires more state-of-the-art studies to provide evidence and bases.

## 1. Introduction

Fractures are common presentations in the emergency department. Among those, proximal humeral fractures are the third most common fractures observed in elderly osteoporotic patients following wrist and hip fractures [1–3], accounting for 4%-5% of all fractures and 45% of total humeral fractures [4]. The incidence of proximal humeral fractures tends to increase in the elderly [5–9] or in patients with previous fractures [10]. In terms of classification, several fracture patterns, including the broadly used AO/OTA classification, have been described [11–14]. However, Neer's classification remains the practical system for clinical decision-making, which was based on the displacement of four segments including head, greater tuberosity, lesser tuberosity, and shaft [14]. After suffering from proximal humeral fractures, various interventions, such as open reduction and intramedullary nailing, open reduction and internal fixation, hemiarthroplasty, and total shoulder arthroplasty, would be applied [15]. However numerous factors have a notable impact on fracture intervention, for example, the characterized features of patients, the radiography manifestations, the concomitant injuries, and the severity of fractures [16, 17]. Despite the intensive care for individual patients, the outcomes and prognoses are still despaired and unfavorable. The mortality rate at one year was 10% and even higher in patients not living in their own home, pursuing recreational activities, being able to perform their own shopping and dress themselves, and receiving surgery 3 days or more after admission due to various reasons, for instance, age of 80 years or older, public insurance and no insurance, low household income, urban hospital, and the presence of polytrauma [18, 19]. Different choices of treatments may lead to different complications [20]. The overall complication rate was very high, and the most common complications were fracture displacement, malunion, screw penetration, and humeral head necrosis, especially in patients with treatment of conventional plate and hemiarthroplasty [15, 21]. Therefore, how to make satisfied surgical outcomes of proximal humeral fracture fixation and figure out which is the optimized treatment for the patient in each individual case becomes the most critical issue.

Finite element analysis (FEA), as a numerical tool, is used for quantification and simulation of structures and systems, providing an accurate prediction of a component's response subjected to different kinds of loads and boundary conditions. The subject-specific finite element (FE) model is constructed based on computed tomography or other images [22]. The constructed model is simplified into small units called elements that are assigned with specific material and structural properties, followed by being analyzed under certain boundary conditions to get the component's response [23]. Generally, key aspects of finite element analysis in orthopedics and traumatology are material modes chosen for bone and implant, mesh of model, boundary and loading conditions, and validation. Brekelmans et al. first introduced finite element method in the investigation of biomechanics in this area [24]. Since then, many studies have been carried out by using finite element method in this area. FEA is an extensively used numerical technique applied to test novel implants or new materials, to investigate strain and stress distribution and load transfer between implants and bones [25–28]. What is more, it has been used to compare different implants in fracture fixation [29, 30], analyze biomechanics of bones [31, 32], and even simulate various kinds of models in animal [33, 34] and sports injuries [35]. FEA can not only help to make subject-specific pre-, intra-, or postoperative assessment, which is prerequisite for successful management, but also help reduce operation time, blood loss, rate of screw penetration, rate of screw loosening, intraoperative injuries, and fracture healing time, leading to successful fixation and anatomical restoration of fractures [36, 37].

Treatments of proximal humeral fractures remain challenging and should take everything into account. A growing number of FEA studies have been applied to proximal humeral fractures. FEA can help us analyze proximal humeral fractures precisely and accurately, simulate the surgical procedures, make personalized plans for patients, instruct the postoperative rehabilitation, and eventually provide evidence for clinical practices. However, no review article concerning the applications of FEA in evaluating proximal humeral fractures has been reported. Therefore, the present article summarized the applications, contribution, and clinical significance of finite element analysis in assessing proximal humeral fractures and reviews how this method has been applied to evaluate proximal humeral fractures.

## 2. Applications

### 2.1. Biomechanics Testing

Most studies of biomechanics were performed on cadavers or synthetic humeri [38–40]. However, cadavers can result in a huge cost, ethic issues, and, more importantly, the harmful effect on the researchers' health due to the chemicals for preservation. FEA can not only avoid those problems but also make a contribution in analyzing biomechanics precisely and accurately with a lower cost [28, 41–46]. Biomechanics of bones play a vital role in understanding fracture patterns, fracture mechanism, complications, and treatments. Knowing the mechanical characteristics of implants and bones is the first step in treating patients successfully.

The FE model of proximal humeral locking plate was constructed by Lin et al., aimed at testing when the proximal humeral locking plate would fail under the condition of 0–120° arm abduction. It was found that stress was concentrated and exceeded the maximum in proximal one-third of the locking plate at 120° arm abduction under the condition without strong bone-to-bone contact in the fracture region. What they found could instruct the postoperative rehabilitation by avoiding 120° arm abduction to keep the implant from failure [41]. Both Su et al. and Chen et al. constructed a FE model of humeral fracture with angle of 30°, 45°, and 90° between the fracture face and axis of humerus to record the stress and strain distribution of humerus. They found that stress in the fracture face was higher than nonfracture face and distributed asymmetrically with the center of the fracture face. Maximum stress was observed in the regions 10 mm away from the fractures line. What they found could provide an instruction for surgeon in choosing suitable implants which could distribute the stress and strain equally in the fracture side to meet different stress distribution [47, 48]. Aiming to improve the efficiency and decrease the costs of FEA, Inzana et al. performed biomechanical test to compare pseudothreaded (a smooth cylinder with the threads) model and bonded model to figure out which one is more close to the behavior of the finely threaded screw model in the single screw-in-bone system under the condition of five loading directions and four Young's moduli. They found that the pseudothreaded model could represent the finely threaded screw model more accurately, avoiding higher computational costs. Furthermore, Inzana et al. made a comparison between pseudothreaded and bonded interface across the three different screw configurations in proximal humerus. They realized that the implant stability is not affected in two bone-screw interface and thus drew a conclusion that bonded interface could serve as a more efficient methodology for proximal humeral fractures with PHILOS plate fixation on account of simplicity and less computational costs [49]. Sometimes the novel implant testing is very difficult. However, FEA provides a crucial platform for evaluating the novel implant before clinical application. Murdoch et al. tested new retractable intramedullary nail (a nail with pins can stabilize the humerus without interlocking screws) using FEA, showing a reliable performance on the aspect of compression and torsion [42].

Most of the proximal humeral fractures happened in elderly patients with osteoporosis [1, 9]. Implant fixation in the osteoporotic proximal humerus is remarkably challenging for surgeons [50–52]. It is better to know the bone density before choosing implants to reach better clinical outcomes. Maldonado et al. found that the strain in fractured osteoporotic bones was considerably higher than that in more healthy bones through FEA. Maximal strain values were found for the intact and fractured bone at 90° arm abduction. The results demonstrated that bone mass should be taken into consideration in surgical treatments of proximal humeral fractures in osteoporotic patients [43]. Clavert et al. studied the distribution of stress in several types of bone models and showed that the stress was higher in osteoporotic bones, indicating that deformation more likely happened in the lower bone mass area. What is more, the stress of cancellous screws used in securing implants increased on osteoporotic areas. They demonstrated that the increase in load and stress distribution for an osteoporotic bone must be taken clearly into consideration in designing osteosynthetic implants for treatment of proximal humeral fractures [44]. Wirth et al. measured 12 finite element models of humerus constructed by computers. Screws were inserted digitally into humeral heads at various positions to test implants' stability. They revealed that not only bone mass but also its arrangement in the trabecular microarchitecture were important for implants' stability [45]. Another two studies of Wirth et al. proved that bone mass and the use of bone augmentation with bone cement for improving implant anchorage in low quality bone had strong impacts on implant stability [28, 46]. All in all, bone density is a significant factor affecting the management of proximal humeral fractures and it is significant to consider bone quality when planning humeral surgery. The applications of FEA in studying bone density can provide proofs for treating osteoporotic patients, such as choosing a suitable implant.

### 2.2. Open Reduction and Internal Fixation (ORIF)

Open reduction and internal fixation have been widely applied in proximal humeral fractures. Internal fixation has historically been achieved through various implants and techniques ranging from transosseous suture fixation and tension-band wiring of fracture fragments to applications of semitubular, buttress, cloverleaf plates and locking proximal humeral plates. Locking proximal humeral plates have been proved to be more stable and reliable, especially in osteoporotic patients [15, 53–58]. However, complications including screw penetration, osteonecrosis, subacromial impingement, and loosening of screws have been reported [15, 54, 59].

A lot of finite element models have been constructed to find the relationship between implants and bones, providing basic knowledge and mechanisms for treatment strategies to decrease complication rate and reach better clinical outcomes.

Feerick et al. conducted a FEA investigation of proximal humeral fractures fixed with locking plate, intramedullary nail, K-wires, and Bilboquet device. And each group was divided into subgroups with or without calcium phosphate cement augmentation. Properties of each device have also been tested. A three-part proximal humeral fracture was created. Fracture fragments displacement and pressure stress distribution as well as shear stress distribution within humeral head were recorded. The results of this analysis revealed that shear stress within humeral head increased with increasing angle of loading, reached a maximum at 90° arm abduction, and reduced again at 120° arm abduction. They also demonstrated that cement reinforcement increased the stability and reduced the magnitude of fracture line opening as well as the shear displacement between the fracture fragments. Another important finding of this study was that stress was higher in osteoporotic cortical bone, indicating a high potential for screw pullout/pushout in those patients [60]. This finding was the same as the previous finding introduced by Maldonado [43].

In clinical practice, surgeon should consider and balance many factors contributing to the implant stability. Both experiment test in vitro and finite analysis test were performed by Stoffel et al. to evaluate factors affecting the stability of locking compression plate for diaphyseal fracture [61]. They denoted that, for fractures of the humerus, predominated with rotation forces, three to four screws on either side should be enough. For comminuted fractures with large fracture gap, placement of the innermost screws was prerequisite for the fixation stability. Furthermore, in order to provide enough axial stiffness, the distance between the plate and the bone ought to be kept small and long plates should be used.

He et al. proposed a FE model of proximal humeral fractures to compare the biomechanical characteristics under fixation with lateral locking plate (LLP) or LLP with a medial anatomical locking plate (LLP-MLP). The construct stiffness, fracture micromotion, and stress distribution on the implants were recorded and compared under compressive and rotational loads. The results showed that LLP-MLP possessed better biomechanics, less fracture micromotion, and higher construct stiffness [62]. In practice, adding MLP can provide stronger support for the medial column and therefore improve fixation stability.

Cukelj et al. developed a FE model to simulate the proximal humeral internal locking system (PHILOS) and Arthrex plates in treating three-part proximal humeral fractures. The static load was added. Total bone displacement and maximum bone displacement in the fracture gap were recorded. The results showed that lower total bone displacement and maximum bone displacement in fracture gap were observed in PHILOS plate, indicating that PHILOS plate provided more stable fixation than Arthrex plate in treating proximal humeral fractures [63]. Hence, the PHILOS plate was more practical and useful in clinical surgeries in three-part proximal humeral fractures.

One of the determinants of successful ORIF is medial column support [64, 65]. Burke et al. demonstrated that better bone stock for medial column support of humerus could reduce complications, especially in patients with osteoporosis [66]. The result was consistent with what Brunner found in a prospective multicenter analysis [54]. Both Burke et al. and Lescheid et al. used synthetic humerus models to compare the efficacies of locking plate fixation with or without medial column support in maintaining the reduction of proximal humeral fractures. They both presented promising results that medial column support provided more stable reconstruction and reduced complications [67, 68]. More importantly, the significance of medial column support was verified by finite element analysis. Yang et al. proposed a FE model of standard three-part proximal humeral fracture to examine the effect of medial cortical support and medial screw support on the stability of fixation. Both medial screw support and medial cortical support decreased maximum stress and a combination of both dramatically decreased the maximum stress. They concluded that placement of medial screws combined with good medial cortical contact provided stability for the fixation of proximal humeral fractures [69]. The study, described by He et al., proved that lateral locking plate with a medial anatomical locking plate possessed better biomechanics, less fracture micromotion, and higher construct stiffness. The result indirectly demonstrated that medial column support can provide significant stability in proximal humeral fractures [62]. All the studies above highlighted the importance of medial column support and instructed surgeon to reconstruct the stability of medial column during surgery.

### 2.3. Intramedullary Nailing

Intramedullary nailing has been applied to treat diverse kinds of fractures for many years, especially for the long bone shaft fractures. For some studies, they claimed that intramedullary devices are biomechanically superior to plates for some unstable subcapital humeral fractures or conservative treatments in young individuals [70, 71]. In one cadaveric study carried out by Rothstock et al., they claimed that intramedullary nailing can provide better stability in fixation of three-part proximal humerus fracture, particularly in the fixation system with additional locking screw-in-screws inserted through the head of the proximal screws and a calcar screw inserted to provide additional medial support [72]. Proximal humeral nailing is minimally invasive, with less soft tissue dissection and more vessels preservation. Intramedullary nailing fixation has achieved good fracture union and satisfactory functional outcomes, particularly in younger patients and those in active employments [73]. However, the complication rate of the intramedullary nailing was 25.8%, reported in a meta-analysis carried out by Wang et al. [74]. Osteonecrosis, reoperation, infection, nonunion, acromioclavicular joint impingement syndrome, and screw cutout have been reported in several studies [74–76].

FEA has potential usefulness as a simulation method for examining biomechanics of intramedullary nailing and the interaction between implants and bones. In the cadaveric study described by Edwards et al., they demonstrated that the early failure of proximal humeral intramedullary nailing was due to the high moment transmitted to the locking proximal screw-bone interface in the implants [58]. The result was confirmed by what Giudice did in both animal bone and FE model. The FE model of proximal humeral fractures with intramedullary nailing fixation was constructed. The study was directed at investigating the stress state arising at the contact between the expansion flanges and the medullary canal, defining the properties of the bone-implant contact zone and quantifying the stresses. The results highlighted that the stress peaks due to the contact were detectable at limited points on the extremities of the flanges and the stress state was very high on the internal part section of bones, which explained why the bone-implant contact was inefficient in the distal expansion system [77]. For the clinical practices, they suggested that the intramedullary nail being chosen should be compatible with the length of the humerus being treated. It was not appropriate to use the intramedullary nail in particularly distal fracture because of the stress peaks in the extremities of the flanges. Feerick et al. conducted a computational FEA investigation of proximal humeral fracture fixed with the intramedullary nail. The results indicated that pressure concentrations occurred in the regions of cortical bone at the tips of the screws and shear stress within the cortical bone of the humeral head which increased with increasing angle of abduction. The highest shear stress occurs at 90° arm abduction, with a reduction observed for 120° arm abduction [60]. The studies of intramedullary nailing using FEA complement the indications and contraindications for management of patients and provide evidence for choosing a suitable intramedullary nail for specific patient.

### 2.4. Hemiarthroplasty and Total Shoulder Arthroplasty

In 1893, Péan, the French surgeon, first described hemiarthroplasty of shoulders [78]. In 1955, the humeral head prosthesis was introduced by Neer [79]. Since then, replacements of humeral head have become gradually recognized, and more and more clinical reports were published. Nowadays, hemiarthroplasty has been largely applied in complex proximal humeral fractures, especially in elderly patients with osteoporosis. Some studies proved that arthroplasty has established promising outcome [80]. However, there are still some undesired complications, such as glenoid notching, tuberosity malunion, prosthetic migration, prosthetic subluxation, rotator cuff lesion, heterotopic ossification, posttraumatic osteoarthritis, tuberosity displacement, or malreduction [21, 81].

Many factors could result in unfavorable complications and instability in replacement therapy. Among those factors, the preservation and stability of tuberosity are most crucial for reducing those compilations and producing promising outcomes [44, 82–86]. Abu-Rajab et al. stated that fixation of tuberosity sutured greater and lesser tuberosity to each other or to the shaft, delivered maximum stability, and minimized potential intertuberosity separation through evaluating the effect on movement in sawbones [85]. The significance of fixation of tuberosity was confirmed by FEA. Zhang constructed FE models of greater tuberosity fractures to compare three kinds of fixations, including screws, tension band, and locking plate. The maximum Von Mises stress and stress distribution were compared in the study. They drew a conclusion that the locking plates for great tuberosity showed more obvious biomechanical stability than the other two fixations because it could disperse the stress more easily and evenly [87]. Clavert et al. proposed a FE model of humerus and rotator cuff muscle to study the distribution of stress. The result revealed that the peak of stress distribution was observed in tuberosity, corresponding clinically to the areas where the tuberosity was torn and fractured [44]. What they discovered helped explain why certain types of osteosynthesis fail were due to tuberosity reconstruction failures. If the tuberosity was placed in an unstressed isolated circumstance, the bone would be absorbed due to the absence of mechanical stimuli, described by Baumgartner after performing a FEA test [88]. What Baumgartner observed corresponded to the theory described by Wolff that if the loading on a bone decreases, the bone will become less dense and weaker due to the lack of the stimulus required for continued remodeling. Both higher and lower stress distribution of tuberosity would lead to failure of fixation. Hence, the applications of FEA in fixation of tuberosity instruct the doctors to pay more attention to reconstructing the tuberosity and balancing the stress distribution of tuberosity during replacement.

What is more, Büchler and Farron reconstructed a FE model of a healthy shoulder to study the influence of the shape of prosthetic humeral head on shoulder biomechanics and to evaluate the benefits of anatomical reconstruction of the humeral head after shoulder arthroplasty. They found that the anatomical reconstructions of the humeral head during shoulder arthroplasty provided stability and had significant effects on pressure and stress distribution in the glenoid of the intact shoulder [32]. Furthermore, Pressel et al. highlighted that bone density was reduced around the prosthesis through FEA because the stresses were transmitted through the prosthesis, leading to low bone stresses surrounding the prosthesis and high stresses distally from the prosthesis. The results could be explained by bone resorption around the prosthesis caused by stress shielding [89]. Another study, conducted by Schmidutz, highlighted stress shielding and bone resorption under the central implant shell in cementless shoulder resurfacing arthroplasty [90]. The finding might remind doctors to pay more attention to the stress distribution during replacement to keep implants from failing and encourage restoring the prosthetic head in anatomical position during replacement.

### 2.5. Augmentation with Bone Cement

Augmentation with bone cement is a common technique used in orthopedics and traumatology to enhance the stability of implant-bone interactions and to reduce failure or complications of fixation. The functions of bone cement have been proved to be effective in clinical cases [60, 91], synthetic humerus studies [39], or cadaveric studies [92–95]. Except for the cadaveric studies, the powerful numerical tool, finite element analysis, also showed great ability to investigate the effects of bone cement augmentation. Kennedy et al. implemented a FE model of three-part proximal humeral fracture fixed with plates, augmented with or without cement. They measured the pressure gradient around the screws, the peak pressure of the fracture planes, and the mean stress of the screws. The result showed that augmentation with cement improved stability and reduced stress at the implant-bone interface [96]. Wirth et al. simulated a FE model to study whether augmentation of peri-implant-bone or osteoporotic bone loss would affect implants stability. They revealed that peri-implant-bone augmentation increased implant stability and the efficiency of bone augmentation decreased with increasing peri-implant distance. What is more, bone mass loss led to a decrease in implant stability [46]. Another FEA study carried out by Feerick et al. also confirmed the importance of bone cement augmentation in management of proximal humeral fractures [60]. For clinical practices, these findings highlight the potential of bone cement augmentation for improving implant anchorage in low quality bone and the use of augmentation may improve clinical outcomes.

## 3. Conclusions and Perspectives

In conclusion, FEA has been applied to investigate various aspects of proximal humeral fractures for a better understanding. Our study summarizes the applications, contribution, and clinical significance of finite element analysis in assessing proximal humeral fractures. Finite element analysis can help us better understand biomechanics, select optimized choices, make clinical decisions, and evaluate management of proximal humeral fractures. FEA can be used as a powerful noninvasive tool to evaluate biomechanic characters of novel implants and simulate surgical procedures for clinical cases, especially the complex proximal humeral fractures for junior doctors. Compared to the studies based on cadavers or clinical cases [38–40, 58, 92–95], FEA can be used as an alternative method for the expensive and ethically sensitive animal or cadaveric testing. Therefore, FEA can provide more specific, accurate, and precise values for clinical practices.

However, for investigation involved in finite element method, the boundary conditions become difficult to handle owing to the sophisticated structure of shoulder joint. It is impossible to simulate the real boundary conditions exactly with all the muscles and ligaments acting together [32, 36, 44, 97]. Most of the studies we reviewed simplified the shoulder joints for investigation by ignoring the interaction of muscles, ligaments, bones, and other surrounding structures. Therefore, for those more complicated boundary conditions, it is better to develop a model that can simulate real condition for a precise investigation of proximal humeral fractures. Besides, obtaining the parameters to design the properties of bones and other surrounding structures remains challenging. For proximal part of humerus, different regions of bone have different properties, for example, Young's modulus and Poisson's coefficients which can affect the simulated effects or outcomes largely [44, 98, 99]. Also, the bone density of the patients should be taken into account when designing the material models. Both elasticity and plasticity should be included in the simulation for a more realistic outcome [100, 101]. Hence, more studies should be carried out to provide more information regarding the properties of simulated material. What is more, most of the FE models we reviewed here were originated from images of healthy individuals rather than the specific patient with distinct fracture. And also the fracture models were created based on the standard fracture classifications with regular fracture lines, regardless of the characteristics of distinguishing patient. Most of the FEA researches were fundamental study rather than clinical research. The results of those studies should be translated into clinical practices and provide evidence for doctors in practice to handle personal patient. In the future, more FE models should be constructed according to the specific fracture pattern of unique patient and translated into clinical practices, which can help clinicians to make personalized interventions for each patient.

## Abbreviations

FEA: Finite element analysis
FE: Finite element
ORIF: Open reduction and internal fixation
LLP: Lateral locking plate
MLP: Medial anatomical locking plate
PHILOS: Proximal humeral internal locking system.
